# Facial Expression Overrides Lumbopelvic Kinematics for Clinical Judgements about Low Back Pain Intensity 

**DOI:** 10.1155/2016/7134825

**Published:** 2016-03-29

**Authors:** A. Courbalay, T. Deroche, M. Descarreaux, E. Prigent, J. O'Shaughnessy, M.-A. Amorim

**Affiliations:** ^1^CIAMS, Univ. Paris-Sud, Université Paris-Saclay, 91405 Orsay Cedex, France; ^2^CIAMS, Université d'Orléans, 45067 Orléans, France; ^3^Département des Sciences de l'Activité Physique, Université du Québec à Trois-Rivières, Trois-Rivières, QC, Canada G9A 5H7; ^4^LIMSI-CNRS, Univ. Paris-Sud, Université Paris-Saclay, 91405 Orsay Cedex, France; ^5^Département de Chiropratique, Université du Québec à Trois-Rivières, Trois-Rivières, QC, Canada G9A 5H7

## Abstract

*Background.* Through real-time behavioral observation systems, pain behaviors are commonly used by clinicians to estimate pain intensity in patients with low back pain. However, little is known about how clinicians rely on pain-related behaviors to make their judgment. According to the Information Integration Theory (IIT) framework, this study aimed at investigating how clinicians value and integrate information from lumbopelvic kinematics (LK), a protective pain behavior, and facial expression intensity (FEI), a communicative pain behavior, to estimate pain in patients with chronic low back pain (cLBP).* Methods.* Twenty-one experienced clinicians and twenty-one novice clinicians were asked to estimate back pain intensity from a virtual character performing a trunk flexion-extension task.* Results.* Results revealed that both populations relied on facial expression and that only half of the participants in each group integrated FEI and LK to estimate cLBP intensity. Among participants who integrated the two pain behaviors, averaging rule predominated among others. Results showed that experienced clinicians relied equally on FEI and LK to estimate pain, whereas novice clinicians mostly relied on FEI.* Discussion.* The use of additive rule of integration does not appear to be systematic when assessing others' pain. When assessing pain intensity, communicative and protective pain behaviors may have different relevance.

## 1. Introduction

It is widely accepted that clinicians usually estimate and manage others' pain by drawing inferences from several pain-related behaviors they perceive [1, 2]. Various actions, including language, paralinguistic vocalizations, and facial expressions (i.e., communicative pain behaviors), but also body posture and escape or avoidance behaviors (i.e., protective pain behaviors) may signal pain to clinicians [3]. These pain behaviors are deeply rooted in real-time behavioral observation systems commonly used by clinicians in order to assess patient's pain [4]. All these systems have common features: they use a standardized test situation to elicit controlled behavioral responding; the sequence in which the tests are carried out is randomized to prevent order effect; trained observers estimate the presence/severity of each behavior characteristic of pain on a two- or three-point rating scale; then examiners count/sum the amounts of pain behaviors coded to provide an overall score reflecting the intensity of the pain experienced by the patient. Such real-time behavioral observation systems are based upon a number of assumptions, including the notion that additive accumulation of behaviors reflects greater pain and that the behaviors resemble one another in their metric properties [4]. To date, there is little evidence regarding these assumptions. Then, the present study has been designed to provide a theoretical and methodological framework to better understand how clinicians judge others' pain based on nonverbal behaviors.

The methodological framework used in the present study was an application of Anderson's Information Integration Theory (IIT) [5]. The basic aim of this theory is to identify the cognitive algebra used to combine different sources of information (here, different pain behaviors at different intensities) for decision-making (here, the estimation of others' pain intensity). Three processes operate between the observable stimuli and the observable response: valuation, integration, and action. Valuation refers to initial processing stages transforming each observable stimulus (Φ) into a psychological representation (Ψ). The integration operator then combines these different psychological representations into an implicit response. This integration is completed through a class of adding, multiplying, or averaging algebraic rules. Finally, the action operator converts the implicit response into the observable response, which, in pain case study, is the observer's rating of others' pain intensity on a visual analog scale (VAS), commonly used in clinical settings. Valuation and integration processes do not necessarily imply observers to have an accurate representation of a situation. IIT main concern is to determine whether observers took into account several sources of information from a situation and how they combine them to judge the situation overall. In the present study, the use of functional measurements conducted within the IIT framework [5] allowed us to (a) infer how clinicians value the different information from pain behaviors when they estimate pain intensity (i.e., valuation function) and (b) account for the psychological laws used to combine both sources of information (i.e., integration function). Functional measurement has been recently used to study pain behaviors assessment [6]. For this purpose, the authors highlighted the notion that clinicians' pain estimates are not always proportional to the increase of pain behavior related intensity. Second, they showed that when estimating others' pain, several rules of integration (i.e., additive-like and multiplying but also averaging) are used by observers, although sometimes only one pain behavior is processed. Back to the validity of real-time observation systems [4], such results highlight the potential shortcoming of some assumptions upon which they have been based. Thus, the purpose of this study is to replicate and extend these earlier results.

Since chronic low back pain (cLBP) constitutes a major public health issue, as more than 85% of patients who suffer from it are diagnosed with LBP from nonspecific origin [7], and since real-time behavioral observation systems have been used to infer pain in this population [8], the present study focuses on the way clinicians rely on two LBP prototypical behaviors to assess pain intensity. It is well recognized that facial expressions of pain, considered as a communicative pain behavior, are commonly used by clinicians to assess pain intensity in patients with health problem in general and with low back pain specifically [4]. Lumbopelvic kinematics (LK) also constitute relevant back pain-related information. LK refers to a simultaneous movement performed in a rhythmic ratio of lumbar movement (L) to pelvic rotation (P), during a complete cycle of trunk flexion and extension. Individuals with cLBP tend to increase lumbar flexion (i.e., having larger L/P ratios compared to healthy individuals) during the early stages (0–30°) of forward bending and decrease lumbar flexion (i.e., having lower L/P ratios) during midrange of forward bending (30–60°) [9]. It has been suggested that such adaptations, often considered protective pain behaviors, are put forward by patients experiencing pain to reduce the threat to spinal tissue and possibly prevent further pain and injuries to the spine [10].

Through the methodological framework of IIT [5], the present study has two main objectives: (1) to examine how clinicians perceive and value information from LK and facial expression intensity (FEI) when estimating cLBP intensity and (2) to question whether the additive rule prevails in their pain estimates. These questions have been addressed in light of clinicians' experience. Indeed, a previous study found that high levels of prior exposure to pain, which is deeply rooted in clinicians' activities, were unrelated to pain expression sensitivity but did significantly diminish the likelihood of judging others to be in pain [11]. In addition, studies showed that expert physicians gave significantly lower patients' pain ratings than did novice physicians, compared to patients' self-reported pain experience [12, 13]. Nevertheless, other studies found that novice therapists showed weakness in their clinical reasoning skills compared to expert clinical reasoning [14] and that experts had a clearer idea as to the patients' possible problems from the start of the physical examination [15]. According to this diverse literature, there are motives to examine how clinical experience (i.e., experienced versus novice clinicians) contributes to the way clinicians rely on pain behaviors.

## 2. Method

### 2.1. Participants

Participants were divided into two groups: 21 experienced clinicians [all chiropractors, i.e., spine specialists: 9 females, 12 males; mean age = 42.24 years, standard deviation (SD) = 12.38; years of practice = 16.4, SD = 10.9] and 21 novice clinicians [all chiropractic interns: 13 females, 8 males; mean age = 23.48 years, standard deviation (SD) = 1.47]. Experienced clinicians were included if they were actively practicing at the time of the experiment. Novice clinicians were included if they were in the fourth or fifth year of their training program at the time of the experiment. If clinicians had experienced an episode of cLBP in the past, they were not included in the experiment. This research project was approved by the Université du Québec à Trois-Rivières Ethics Committee and all participants gave their written informed consent.

### 2.2. Apparatus and Stimuli

The current study examined the visual integration of two pain information sources originating from either the body (i.e., trunk flexion and extension) or the face (i.e., facial expression) when estimating cLBP intensity. Stimuli consisted of a set of 3D realistic characters performing a trunk flexion-extension task, with an avatar imported from the Poser 8® software library.

**Figure 1:**
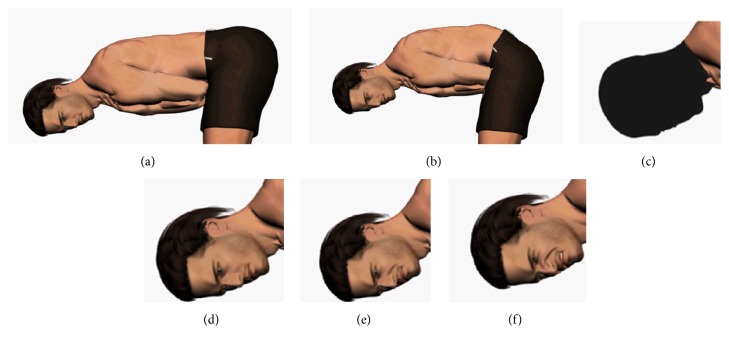
(a, b) Examples of trunk flexion frames taken from a video: (a) illustrates the prototypical low back pain lumbopelvic kinematics (“LBP LK”), whereas (b) represents the prototypical healthy lumbopelvic kinematics (“healthy LK”). (c–f) Examples of facial expression intensity (FEI): (c) corresponds to the masked FEI (“masked FEI”), (d) to “FEI 0%,” (e) to “FEI 50%,” and (f) to “FEI 100%.”

For each stimulus, LK and FEI were manipulated. To begin with, two realistic movements of the character's trunk flexion and extension were created. The LBP LK was developed to simulate a typical guarding behavior observed in patients with LBP [9] (i.e., increased hip flexion and decreased lumbar flexion during midrange of forward bending) whereas prototypical healthy LK was developed using kinematics data derived from healthy adults. Different facial expressions were then associated with each avatar's back pain condition. According to previous studies [16–18], three facial actions were targeted for modeling pain expression: brow lowering (AU4), orbit tightening (AU6&7), comprising “cheek raise” (AU6) and “lid tightening” (AU7), and levator contraction (AU9&10) including the effects of “nose wrinkling” (AU9) and “upper raise” (AU10). The degree of mobilization related to the facial actions' contraction was modulated according to different intensities. The created FEI varied from a “neutral expression” to “maximal pain expression” (i.e., 0%, 50%, and 100%). The unfolding of each facial expression was linear (i.e., constant increases in intensity) and was generated using morphing. Three stimuli were then created, each mobilizing the three typical pain AUs simultaneously at different intensity levels. Following the IIT, in order to obtain a reference relative to the observer's judgments about kinematics only, and to test the averaging rule for information integration, an avatar presenting masked FEI was also created (i.e., masked FEI condition). Consequently, four video stimuli were created for each of the two flexion-extension movements (see Figure 1). All videos began with the character standing up, with a neutral facial expression (i.e., FEI 0%). Then, for each kinematic condition, the FEI was manipulated as follows: FEI stayed neutral (0%) until 30% of the trunk flexion was reached, from which it increased linearly reaching 0% (FEI 0%), 50% (FEI 50%), or 100% (FEI 100%), and decreased linearly when 30% of the extension was reached. Videos were played at constant speed, to limit participants' judgment being influenced by this variable. Each video lasted 10 seconds, in order to correspond to the real duration of a trunk flexion-extension task performed by a person with low back pain [19].

### 2.3. Measures

*Others' pain intensity* was measured via a computerized 100 mm visual analog scale anchored by no pain at all (left side) and the most intense pain imaginable (right side).

### 2.4. Procedure

The experiment began with one block of eight practice trials during which all stimuli were displayed. This practice block, completed prior to the beginning of the experiment, allowed participants to familiarize themselves with the stimuli and the way of rating their answers on the response scale. During this block, participants did not receive feedback with regard to task accuracy. In order to avoid desirability effects, the experimenter could not see the participants' answers but was standing nearby in case of need for precision. Following these practice trials, participants watched two blocks of 16 random video trials, each video being presented four times. Videos (1000 × 748) were displayed at the center of a large screen (1024 × 768 pixels) positioned at a comfortable distance (about 57 cm). The instructions were the following: “You will see an individual who has been experiencing low back pain for the past six months perform trunk flexions and extensions. Observe the person as a whole. After each trial you will have to judge the intensity of low back pain experienced by this individual.” After each video, a 10 cm visual analog scale, only anchored from no pain to maximal pain, was displayed at the bottom of the screen. Participants had to indicate with a mouse click the pain intensity they perceived, which automatically triggered the next trial.

### 2.5. Data Analysis

First, retest reliability values were performed in order to attest that the clinicians' judgment was reliable (see Table 1). Second, classical repeated-measures analyses of variance (ANOVAs) were performed in order to determine the contribution of LK and FEI to the participants' pain estimates. Then, functional measurement analyses were realized with two aims: (1) to examine how clinicians valued and integrated information from LK and FEI when estimating pain intensity and (2) to identify within- and between-group differences in the subjective internal scale range for each pain behavior.

**Figure 2:**
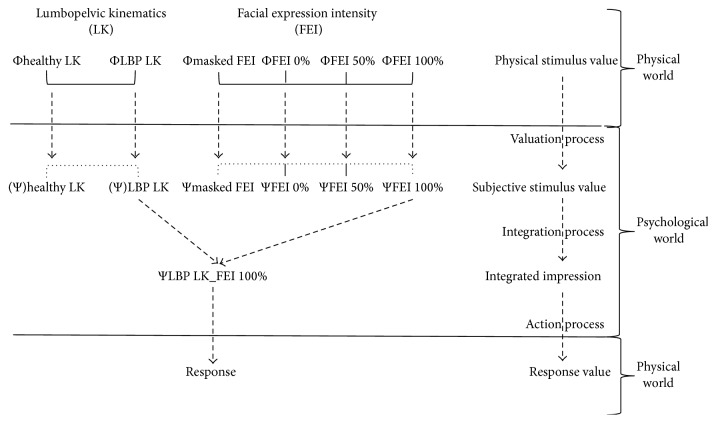
Illustration of Anderson's [5] Information Integration Theory applied to this experiment: from stimulus presentation to participants' response.

**Figure 3:**
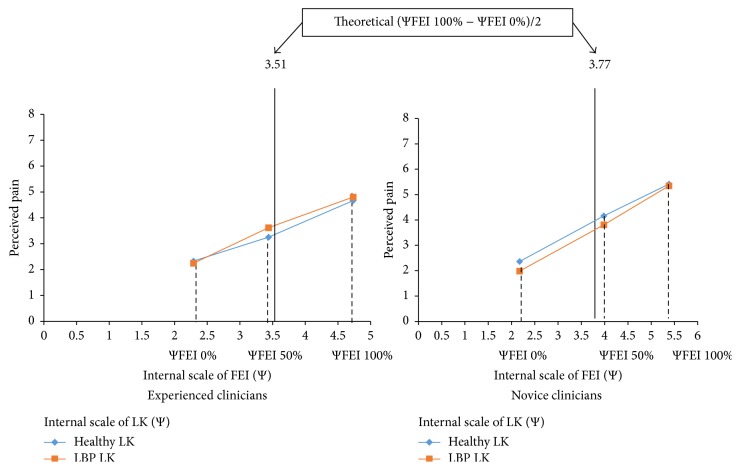
Perceived pain as a function of the internal scale of facial expression intensity (FEI) and of lumbopelvic kinematics (LK), for each group.

Functional measurements propose three processes, corresponding to valuation, integration, and action (response production) processes, illustrated in Figure 2. The valuation process corresponds to the transformation of each physical stimulus value (e.g., ΦFEI 50%) into a subjective internal value (e.g., ΨFEI 50%) mapped on the VAS. Internal values (Ψ) are approximated by the marginal means of responses given by participants for each physical ΦLK or ΦFEI condition. For example, experienced clinicians estimated pain intensity for ΦFEI 50% through two sets of stimuli: Φhealthy LK FEI 50% (mean = 3.25) and ΦLBP LK FEI 50% (mean = 3.62). Therefore, the corresponding subjective internal value ΨFEI 50% (ΨFEI 50% = 3.43) represents the marginal mean of Φhealthy LK FEI 50% and ΦLBP LK FEI 50% pain estimates (see Figure 3). First, analyses were conducted on this process in order to examine how clinicians perceived the magnitude of changes (participants' subjective internal scale) in LK and facial expressions when estimating cLBP intensity.

The integration process was then used to combine the different subjective values into an internal response. Cohen's criterion [20] was used to determine how participants integrated LK and FEI. According to Cohen's *d* formula (*d* = *M*_effect_/SD_effect_), an effect is negligible if its *d* value is lower than 0.2 (i.e., if the mean effect is less than 1/5 of its associated standard deviation). Thus, the effect of LK and FEI was considered to be not negligible if the mean effect was greater than 1/5 of its standard deviation. We used Cohen's* d* formula (*d* = mean effect/standard deviation effect) and his criterion in which an effect is small if *d* value is inferior to 0.20 to define an effect (i.e., factor's internal scale range) as negligible. According to this formula, the effect of LK and FEI was considered to be not negligible if the mean effect was greater than 1/5 of its standard deviation. In the first step, the internal scale ranges associated with LK and FEI (i.e., ΨFEI 100% marginal mean–Ψ 0% marginal mean; ΨLK LBP marginal mean–Ψhealthy LK marginal mean) were, respectively, calculated for each subject. The marginal mean equals the mean of responses given for each physical ΦLK or ΦFEI condition. For example, ΨFEI 100% marginal mean refers to two sets of stimuli Φhealthy LK FEI 100% and ΦLBP LK FEI 100%. In the second step, LK and FEI respective marginal means and associated standard deviations were computed for the whole sample. Then, Cohen's* d* was calculated from these means and standard deviations. The mean FEI internal scale range of all participants was 2.93 (SD = 2.05). Thus, for each subject, the effect of FEI was considered significant if FEI internal scale range (Ψmax–Ψmin) was higher than 0.41 (i.e., 2.05 × 0.2 = 0.41) at the individual level. The same criterion “0.41” was used for LK, although it was more severe than the computed value for LK (i.e., .09 × 0.2 = 0.18). Thus, a second set of analyses was conducted on this process in order to examine how clinicians integrated information from LK and facial expressions when estimating cLBP intensity. Each participant's integration process was studied through the number of variable(s) integrated and from visual inspection of the factorial graphs on an individual-subject basis in order to identify specific patterns of information integration. Participants showing integration of two pain behaviors were classified into an “integration pattern” category which could comprise additive (parallelism pattern), multiplying (fan pattern), and equal weight averaging (parallelism of LK *∗* FEI conditions together with a crossover line for the masked FEI condition) or differential weight averaging (nonparallelism of LK *∗* FEI conditions together with a crossover line for the masked FEI condition) algebraic rule. If only one source of information, or none, was used for judgement (following the abovementioned Cohen criterion), participants were classified under a “no integration pattern.” When participants integrated both LK and FEI,* t*-tests were performed on their data in order to compare the LK and FEI internal scale ranges within groups and identify on which pain behavior each group relied more to estimate LBP intensity.

## 3. Results

### 3.1. Classical Statistics

**Figure 4:**
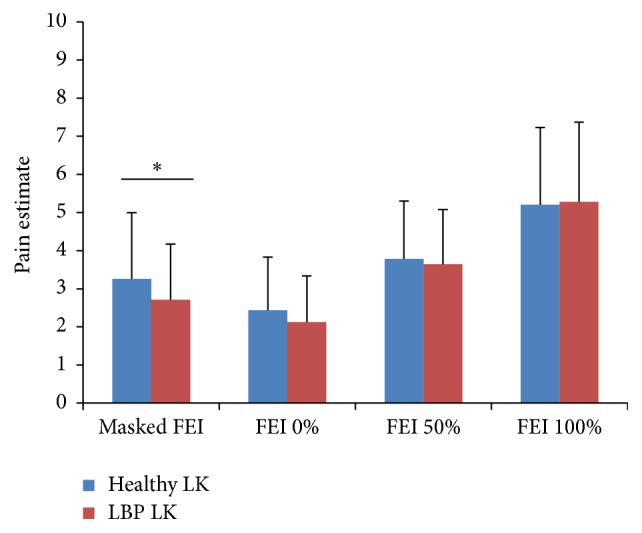
Means and standard deviations for each level of facial expression intensity (FEI) and lumbopelvic kinematics (LK). Only significant LK effects are illustrated with a star (see the text for details regarding FEI effects for each level of LK conditions). Note: ^*∗*^ *p* < .05.

A mixed model 2 × 2 × 4 ANOVA on pain estimates was conducted with groups (clinicians versus interns) as between-subjects factor and with LK (LBP versus healthy) and FEI (masked FEI versus FEI 0% versus FEI 50% versus FEI 100%) as within-subjects factors. There was a significant main effect of FEI *F*(3, 120) = 67.15, *p* < .01, *η*_*p*_^2^ = .63, on judgment and no significant main effect of LK *F*(1, 40) = .56, *p* > .05. Tukey's post hoc analyses showed that the more the FEI was mobilized, the more painful it was perceived, except for the masked FEI condition, which was perceived to be more painful than the FEI 0% condition [resp., *M*_FEI 0%_ = 2.23, *M*_masked FEI_ = 3.03, *M*_FEI 50%_ = 3.71, and *M*_FEI 100%_ = 5.06]. Results also revealed a significant FEI × LK interaction, *F*(3, 120) = 2.88, *p* < .05, *η*_*p*_^2^ = .07, illustrated in Figure 4. Tukey's post hoc analyses revealed a significant LK effect only for the masked FEI condition, *M*_healthy  LK  masked  FEI_ = 3.23, *M*_LBP  LK  masked FEI_ = 2.83. In addition, post hoc analyses showed that pain estimates of FEI conditions differed between each other within each level of LK [for healthy LK, *M*_FEI 0%_ = 2.35, *M*_masked FEI_ = 3.23, *M*_FEI 50%_ = 3.71, and *M*_FEI 100%_ = 5.04; for LBP LK, *M*_FEI 0%_ = 2.11, *M*_masked FEI_ = 2.83, *M*_FEI 50%_ = 3.71, and *M*_FEI 100%_ = 5.07]. Result did not reveal a main group effect, *F*(1, 40) = .82, *p* > .05. In addition, neither the group × LK interaction (*F*(1, 40) = 1.39, *p* > .05), the group × FEI interaction (*F*(3, 120) = 1.46, *p* > .05), nor the group × LK × FEI interaction (*F*(3, 120) = 1.07, *p* > .05) was significant in terms of others' pain intensity. More precisely, each group showed a main effect of FEI [experienced clinicians *F*(3, 60) = 29.36, *p* < .001, *η*_*p*_^2^ = .59; novice clinicians, *F*(3, 60) = 37.98, *p* < .05, *η*_*p*_^2^ = .65)] and no effect of LK, nor of FEI × LK (all *p* > .05). Regarding the latter result, despite the absence of significant LK *∗* FEI interaction at the group level [experienced clinicians, *F*(3, 60) = 1.75, *p* = .17; novice clinicians, *F*(3, 60) = 2.17, *p* = .10], it should be noticed that it reached significance when pooling data from both groups (see above).

### 3.2. Functional Measurement

Figure 3 illustrates functional measurement data of participants' responses expressed as a function of their subjective scaling ΨFEI 0%, ΨFEI 50%, and ΨFEI 100%, corresponding to each physical stimulus value ΦFEI 0%, ΦFEI 50%, and ΦFEI 100% of FEI. Regarding the valuation function, data suggest that, for experienced clinicians, the subjective representation Ψ of ΦFEI 50% lies in the middle (49%) of their internal scale (i.e., between Ψmin = 2.3 and Ψmax = 4.7). In a similar way, for novice clinicians, the subjective representation of ΦFEI 50% is located at 53% of their internal scale of FEI (i.e., between Ψmin = 2.17 and Ψmax = 5.4).* t*-tests confirmed that experienced and novices clinicians did not under- or overestimate FEI scale step (with respect to their internal scale limits Ψmin and Ψmax) for a physical value Φ = FEI 50%, respectively, *t*(20) = .24, *p* > .05 and *t*(20) = .67, *p* > .05.* t*-tests also revealed that the differences between the subjective representation of ΦFEI 50% and the physical value ΦFEI 50% were not different between experienced and novice clinicians, *t*(40) = 1.36, *p* > .05.

**Figure 5:**
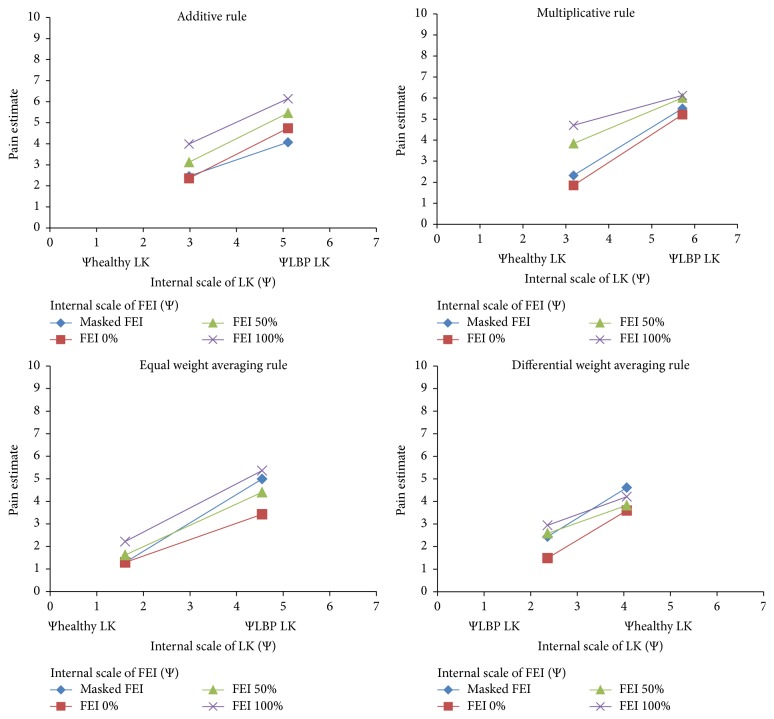
Illustrations of individual differences in participants who integrated both lumbopelvic kinematics (LK) and facial expression intensity (FEI). The functional data of participants' responses is expressed as a function of their subjective scaling Ψhealthy LK and ΨLBP LK, corresponding to each physical stimulus value to Φhealthy LK and ΦLBP LK.

Regarding the integration function, functional analysis revealed that only 13 clinicians among 21 and 12 interns among 21 integrated both LK and FEI (see Table 2). In order to compare the distribution of algebraic rules among clinicians who integrated information from the LK and FEI, a chi-square analysis was conducted with the whole sample (sample sizes were too small to conduct one analysis per group). Given that there was no expectation regarding the distribution of algebraic rule among participants, expected samples were consistent. The chi-square analysis did not show any algebraic rule to predominate among others, *χ* ^2^ = 7.16, *p* > .05 when considering the equal weight and differential weight averaging rules separately. See Figure 5 for an illustration of the integration patterns for each different type of algebraic rule used by the clinicians. However, taken altogether, the averaging rules (including both equal and differential weights averaging models) predominated among others (*n* = 16).

Following functional measurement,* t*-tests were then performed on the data of participants who integrated LK and FEI in order to compare the LK and FEI internal scale ranges. The analyses revealed that clinicians did not present a larger FEI internal scale range (*M* = 2.21) than LK internal scale range (*M* = 1.24), *t*(11) = 1.88, *p* > .05. In contrast, novice clinicians showed a larger FEI internal scale range (*M* = 3.69) than LK internal scale range (*M* = 1.08), *t*(10) = 3.97, *p* < .05. In other words, experienced clinicians who integrated both LK and FEI relied equally on FEI as on LK to estimate cLBP intensity, whereas novice clinicians relied more on FEI than on LK.

## 4. Discussion

According to Prkachin et al. [2], individuals in pain often show behavioral changes that are quite distinctive to observers. As such, reliable and valid real-time behavioral observation systems have been developed aiming at better assessing patients' pain. However, little is known about how clinicians perceive and value information from pain-related behavior and how they integrate them to make their judgment. Thus, according to the IIT [5], the present study provides a theoretical and methodological framework to address this issue. Specifically, we examined the potential contribution of LK and FEI when estimating cLBP intensity, and we studied whether the additive rule prevails in clinical judgements.

First, classical statistics showed that FEI contributed to clinicians' pain-related judgements, whereas they failed to demonstrate that information from LK significantly contributed to clinicians' cLBP pain estimates. IIT brought about additional insights, as it examined how* each* clinician valued and integrated information from LK and FEI to estimate patient's back pain. Individual analyses revealed that 13 experienced clinicians of 21 and 12 novice clinicians of 21 integrated FEI and LK. This result highlights the need to consider other theoretical and methodological approaches such as Information Integration Theory to have a more fine-grained analysis of the cognitive processes that underlie clinicians' judgements of others' nonverbal pain behaviors. Although LK is related to pain experience [10] and constitutes a relevant patient's LBP cue, it does not seem to be systematically accounted for in clinicians' estimates of patients' pain intensity. When assessing a patient's clinical status, LK may be more relevant to assess LBP disability rather than pain intensity. Indeed, LK is already considered a relevant input in evaluating spinal loads [21] and discriminating between low back pain and asymptomatic populations [9, 22]. This suggests that although LK represents a typical pain behavior in cLBP [2], clinicians rely on other pain behaviors to assess their patients' pain intensity. As a result, the contribution of FEI to others' pain estimates is consistent with the literature [1, 23] and reinforces the contribution of FEI to others' pain responsiveness. Given that facial expressions of pain rapidly communicate information about the internal state of an individual in pain to observers, FEI are already recognized as a particularly important channel when judging someone in pain. Overall, this first series of results based on the IIT shows that even though numerous cLBP related behaviors are present in real-time behavioral observation systems, all are not systematically used by clinicians to infer patient's pain.

Functional measurements also showed that, among the twenty-five clinicians who integrated FEI and LK, averaging rules as a whole (i.e., equal weight and differential weight) predominated compared to the additive and multiplying rules. These results contrast with real-time behavioral systems, which assume that accumulation of pain behavior reflects greater pain, suggesting the use of an additive rule [4]. Thus, this assumption seems to differ from clinical practice and might be slightly modified.

In addition, functional measurements conducted on participants who integrated LK and FEI showed that experienced clinicians relied equally on FEI as on LK to infer back pain, whereas novice clinicians relied more on FEI than on LK. These results suggest that novice clinicians are more sensitive to communicative pain behaviors than experienced clinicians. This second result emphasizes another limit of real-time behavioral systems [4]. While those systems argue that pain behavior presents similar metric properties, it seems that when being assessed, pain behaviors may not have the same relevance for observers.

Beyond these results, the current study presents several limitations that should be considered. Indeed, according to the real-time observation systems, pain can be estimated through different typical behaviors, that is, facial expressions and body posture as studied here, but also from verbal and paraverbal expressions [1, 3]. In that experiment, only selected information was given about the patient's history. Yet, these pieces of information are known to participate in the clinicians' judgment elaboration. In addition, previous studies showed that low back pain leads to slower and less intense walking patterns [24, 25]. In the present experiment, it was voluntarily chosen to play all videos at constant and similar speed, in order to focus on lumbar kinematics. All these elements could explain why 50% of the participants only integrated LK and FEI. Moreover, judgment about a person's pain also results from an interaction between an individual in pain and an observer [26]. In our experiment, the avatar characteristics were not manipulated. Yet, the patients (individuals in pain) characteristics, for example, sex [27], age [28], or attractiveness [29], are recognized to influence health care perceptions of individuals with pain. For instance, Hadjistavropoulos et al. [29] found that, compared to low level of physical attractiveness, high levels of physical attractiveness were associated with lower physician pain ratings, distress, need for help and negative affect, and higher physician health ratings. However, given that the main aim of the present paper was to focus on how observers value and integrate information from pain behaviors, it was a deliberate choice to focus on pain behaviors instead of patients' characteristics. In addition, participants were asked to make only one judgement (i.e., pain intensity) for each situation. Nevertheless, an examination of additional pain-related judgements (e.g., level of disability) would have contributed to a better understanding of the results. For instance, it would be interesting to determine whether clinicians utilize FEI as a proxy of pain intensity but use LK as a proxy of functional disability. In a certain way, the use of virtual realistic characters differs from real patients. Despite being not real patients, virtual characters are recognized as a reliable tool [30, 31], recently used in studies investigating others' pain assessment [6, 32, 33].

Notwithstanding these limitations, the present study highlights the potential implication of using IIT framework in order to better understand how clinicians elaborate pain-related judgements from nonverbal pain behaviors.

## Figures and Tables

**Table 1:** Cronbach's alphas related to clinicians' answers per condition.

	Healthy LK	Low back pain LK
Masked FEI	.75	.86
FEI 0%	.89	.83
FEI 50%	.82	.84
FEI 100%	.91	.96

**Table 2:** Distribution of integration patterns across participants.

	Clinicians
	*N* = 42
Additive	8
Multiplying	1
Equal weight averaging	6
Differential weight averaging	10
No integration	17

“Integration” patterns (i.e., additive, multiplying, equal weight averaging, and differential weight averaging) include experienced and novice clinicians who integrated facial expression intensity (FEI) and lumbopelvic kinematics (LK) both. Participants who integrated only one pain behavior (FEI or LK) or less are part of the “no integration” group.

## Additional Points

This study provides a theoretical and methodological framework to examine how novice (*n* = 21) and experienced (*n* = 21) clinicians value and integrate information from facial expression intensity (FEI) and lumbopelvic kinematics (LK) to estimate chronic low back pain (cLBP) intensity. Results indicated that both populations rely on facial expression signals but that only half of the participants integrated both sources of information to estimate cLBP intensity. Contrary to what real-time behavioral observation systems assume, information from pain behaviors was not systematically added by clinicians when making judgments, and novice clinicians were more sensitive than experienced clinicians to facial signals.
